# Novel application of vinylpolysiloxane hearing aid impression mold as patient‐specific bolus for head and neck cancer radiotherapy

**DOI:** 10.1002/ccr3.2731

**Published:** 2020-03-23

**Authors:** Anne Elizabeth Gunter, John Burgoyne, Min Park, Namou Kim, Daliang Cao, Vivek Mehta

**Affiliations:** ^1^ Department of Radiation Oncology Swedish Cancer Institute Seattle Washington; ^2^ Present address: Department of Otolaryngology Madigan Army Medical Center Tacoma Washington

**Keywords:** adjuvant radiation, bolus, head and neck surgery, radiotherapy planning, squamous cell carcinoma

## Abstract

Hearing aid impression material composed of vinylpolysiloxane is an ideal bolus material which may be used to aid in delivery of adjuvant radiation to complex surgical defects of the head and neck. It is affordable, easily accessed, and efficient.

## Graphical abstract

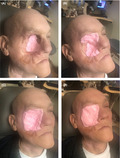

## 1 INTRODUCTION

Administration of radiation in the head and neck cancer patient can present with its own set of unique challenges and dilemmas. The goal of sparing critical structures, while still attempting to deliver therapeutic doses to target tissues, may be limited by tumor location, patient anatomy, and postsurgical defects. Treatment of superficial lesions in the head and neck is a particular obstacle because of the inherent contour irregularities which impact the ability to deliver a consistent dose of radiation. A bolus is a tissue equivalent material used to overcome irregular surfaces and provide a buildup of dose to the surface receiving the radiation therapy. Custom‐shaped boluses have successfully been applied in the treatment of postmastectomy, paraspinal muscle, and head and neck defects.^1^, ^2^, ^3^ Three‐dimensional (3D) printers and computer‐driven milling machines are popular methods utilized to create a customized bolus which conforms to the patient in a way that would alleviate any air gaps.^4^, ^5^ Although gaining popularity, these techniques require special equipment, can be costly, and may take days to produce a customized bolus.

We present a case of a 71‐year‐old man with advanced squamous cell carcinoma of the maxilla with extension to the orbit and overlying skin who underwent composite craniofacial resection and orbital exenteration without reconstruction. A bolus material was needed to help deliver adjuvant radiation therapy to this large, irregular surgical defect of the midface and orbital apex. Vinylpolysiloxane, a silicone elastomer traditionally used to craft ear impressions for hearing aids, was adapted to the defect and served as a consistent prosthetic throughout the patient's 6‐week course of radiation. Here, we describe a novel method of fabricating a bolus for head and neck therapy that is both cost‐effective and efficient.

## 2 CASE REPORT

A 71‐year‐old man presented with a slowly enlarging, cutaneous lesion of his right cheek of several years' duration. A CT scan demonstrated a 7.5 cm mass of the right maxilla with infiltration into the soft tissue of the right cheek, extension into the masticator space, temporalis muscle, and the extraconal fat of the right orbit with destruction of the inferior and medial orbital wall. A biopsy of the lesion was consistent with squamous cell carcinoma. He underwent composite craniofacial resection with maxillectomy and orbital exenteration and was found to have tumor extension well into the infratemporal fossa, pterygopalatine fossa, and lateral wall of the nasopharynx. A major reconstructive effort was not undertaken so as to avoid covering residual tumor and to allow for expeditious healing in anticipation of likely chemoradiation. His final pathology was consistent with squamous cell carcinoma measuring 4.6 × 3.5 × 2.6 cm in size and invading into the orbit, buccal mucosa, skin of the cheek, and skeletal muscle. There was neither perineural nor lymphovascular invasion noted. Negative margins were obtained except on the deep aspect of the resection which had detached fragments of tumor, therefore limiting evaluation. There were no involved lymph nodes. His final pathologic staging based on the American Joint Committee on Cancer (AJCC) 7th edition staging system was T4aN0M0.

The patient had an uneventful recovery and met with radiation oncology and hematology oncology for discussions regarding adjuvant therapy. Following extensive discussions at our multidisciplinary tumor board, and with the patient, the decision was made to undergo active surveillance. Unfortunately, three months postoperatively, the patient had evidence of early local recurrence. He underwent treatment with four cycles of cisplatin/docetaxel with a good clinical and radiographic response. Although the site of his previous index tumor and surgical defect presented unique challenges, multidisciplinary recommendations were to proceed with adjuvant consolidative radiotherapy.

**Figure 1:**
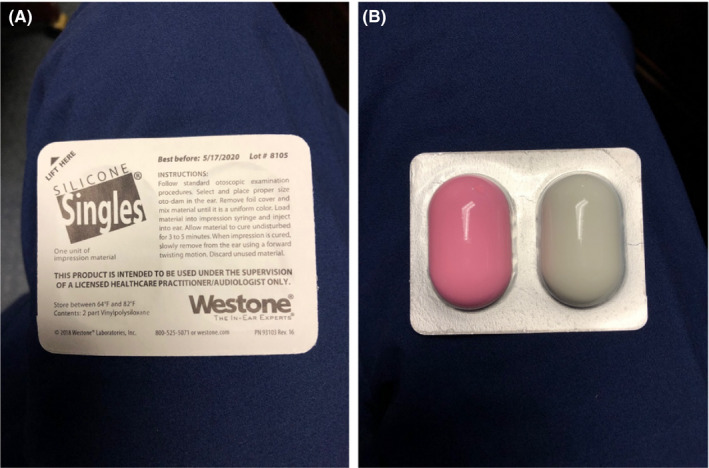
A, packaging from the manufacturer of Silicone Singles^®^ composed of 2‐part vinylpolysiloxane. B, each component of the silicone material is mixed together prior to creating the impression

**Figure 2:**
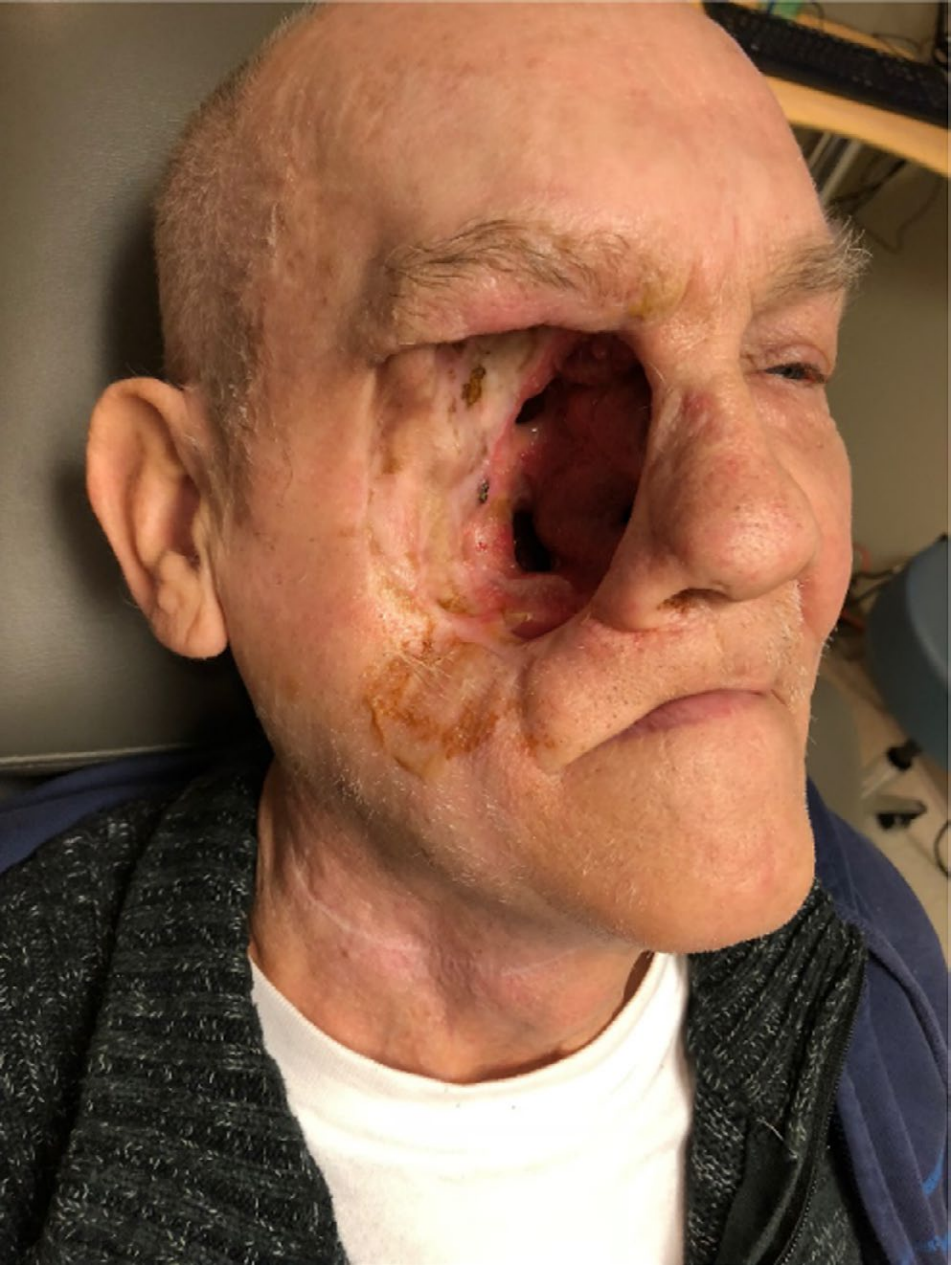
The orbital exenteration and maxillectomy surgical defect posed unique challenges for delivering external beam radiation therapy

**Figure 3:**
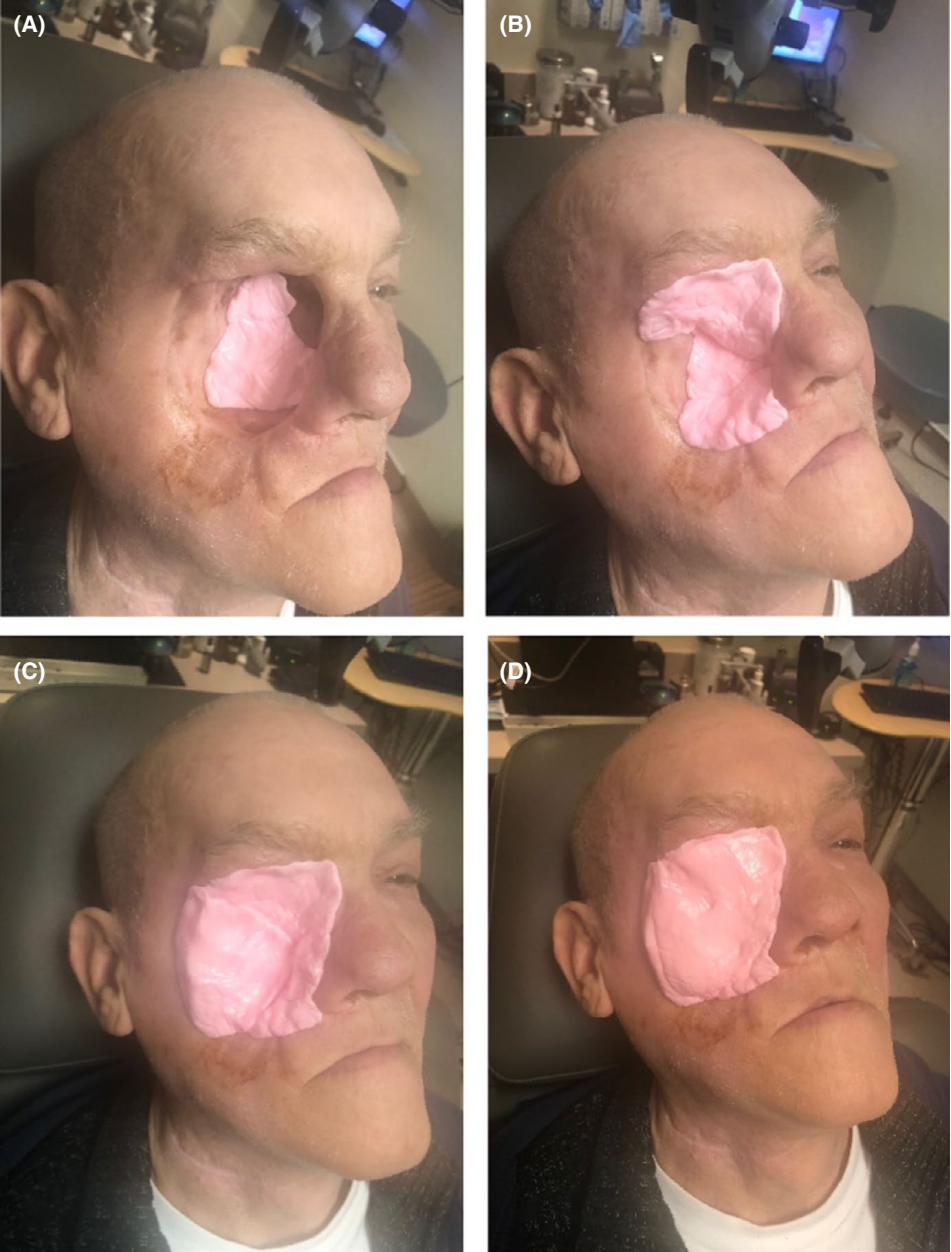
A‐D, the silicone molding is gradually built into the defect to act as a space‐filling device

Initial simulation using gel in the right facial cavity did not create a uniform fit. Silicone Singles**^®^** from Westone (Figure 1), traditionally intended to craft ear impressions for hearing aids, was trialed as an alternative space‐filling device. The substance, known as vinylpolysiloxane, is packaged in two parts and costs $31.00 for a package of 24 units according to the vendor's website. When the components are mixed, the material hardens into a firm yet flexible silicone mold. Eight units were needed to gradually fill the defect and create the final prosthetic to be used as a bolus (Figures 2, 3). This was accomplished during a single clinic visit in the matter of 15 minutes. Planning was conducted, and treatment was undertaken from November 2018 to December 2018. We used volumetric arc therapy (VMAT) utilizing two treatment arcs. The fields and doses used were considered a standard. The tolerances of the critical normal structures were observed. The patient underwent concurrent chemoradiation receiving a total of 60 Gy over 30 fractions. During treatment, the prosthetic was removed and tissues evaluated by an otolaryngologist, then reinserted. Throughout the course of treatment, the prosthetic was worn at all times with tape used overnight to prevent it from falling out. The patient was able to perform all activities of daily living, to include showering, without discomfort. On two occasions, the prosthesis fell out while the patient was sleeping, but it was replaced in clinic without issue. At one point, the patient lost the prosthetic and a new one was replicated with similar ease as described above. The total cost to create the prosthesis and the replacement was estimated to be about $20.00. After the initial replacement, the prosthetic remained in place throughout the course of adjuvant radiotherapy without causing any evidence of infection or inflammation. It was removed at the completion of therapy. The radiation toxicity observed was an expected degree of mucositis in the tissues treated. The side effects were all manageable. The patient tolerated the treatment without any unplanned interruptions in care and remained an outpatient for his entire treatment course.

## 3 DISCUSSION

**Figure 4:**
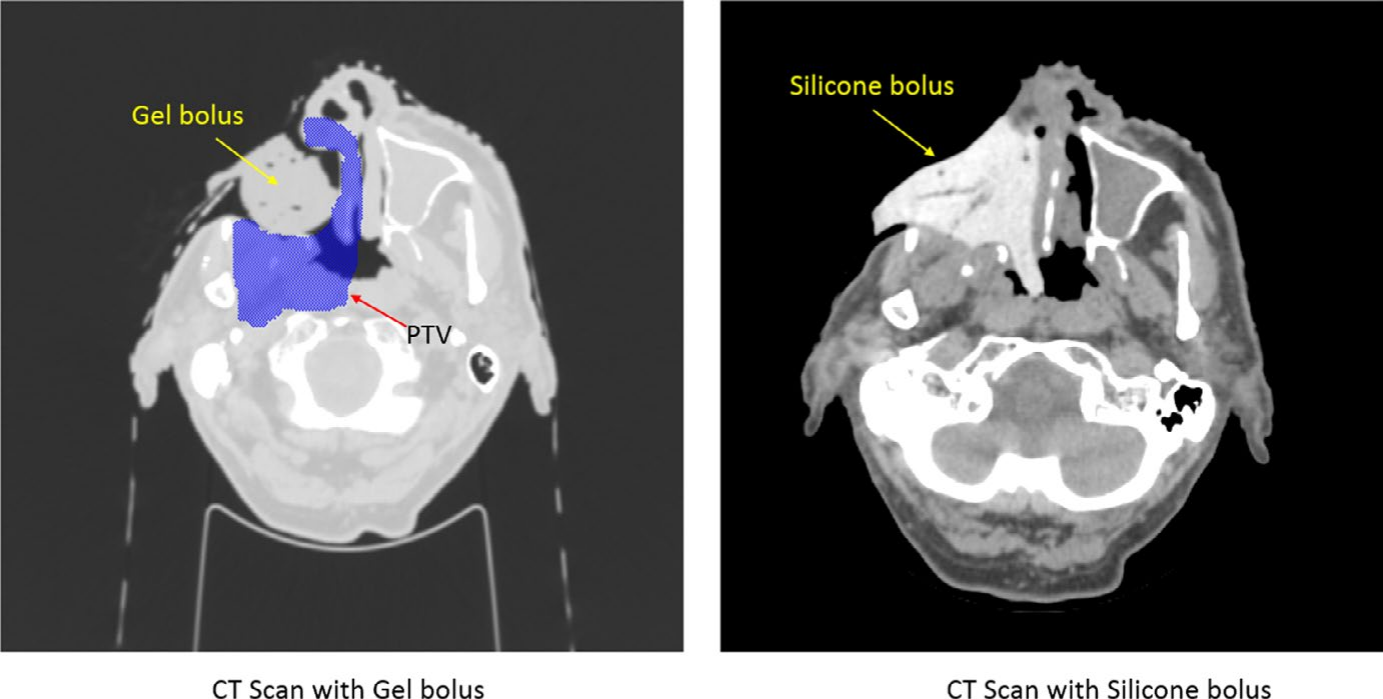
Comparison of the CT scans of the patient using different bolus materials. The left panel uses liquid gel with food wrap film; the right panel uses silicone. As shown in the left panel, the target includes the inner surface of the cavity

**Figure 5:**
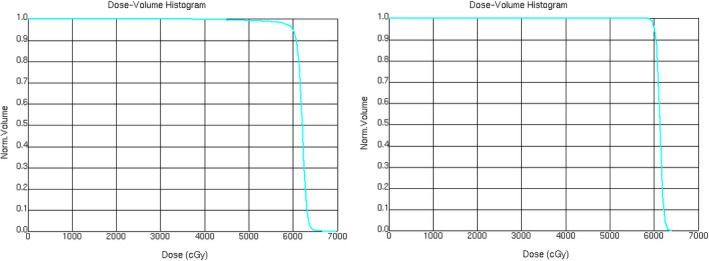
DVH comparison of the volumetric arc therapy plans generated for CT scans using different bolus materials

The unique defect that remains after craniofacial resection with orbital exenteration is a challenge for the radiation oncologist for multiple reasons. First, the variable surface contour creates an uneven dose distribution of radiation. Bolus material, which is placed directly over or within the defect, helps distribute the radiation dose homogenously. However, finding a bolus material that is capable of conforming to surface irregularities of the orbital apex, contralateral nasal side wall, and nasopharynx while eliminating air gaps is an obstacle. As shown in Figure 4, silicone bolus conforms to the inner surface of the cavity much better than gel bolus, leaving almost no air gaps between the bolus and the inner surface. This also led to a better target dose coverage using silicone bolus as shown in Figure 5. In fact, the V95 (defined as the target volume receives at least 95% of the prescribed dose) for the plan using gel bolus is 98.1% and that value increases to 99.9% for the plan using silicone bolus. The standard deviation of the planning target volume (PTV) dose decreased from 9.13 cGy/fraction in the gel bolus plan to 2.52 cGy/fraction in the silicone bolus plan, indicating a more uniform target dose in the silicone bolus plan.

The ideal bolus material is capable of conforming to an irregular contour, is readily available, and inexpensive. It should be resilient enough to withstand ionizing radiation yet comfortable and safe for the patient.^6^ Common materials employed include synthetic gel sheets (eg, SuperFlab), wet gauze, wax, and moldable thermoplastic sheets. SuperFlab is uniform in thickness and flexible, but it does not easily conform into highly irregular concavities. Wet gauze would require repeated packing with each treatment, and the density of the gauze varies with the amount of water used thereby introducing inconsistencies with each treatment.^7^ Thermoplastic sheets are highly shapeable and possess ideal tissue equivalent properties, rendering them useful as bolus materials.^8^ These materials often go through a molding process which requires heating followed by a period of cooling to allow it to set in the desired configuration. Customized boluses composed of modeling wax are frequently described in the literature; however, they are commonly fabricated using a milling machine which can take hours.^1^, ^2^ Both thermoplastic materials and wax are rigid, which is easily tolerated on external surfaces but would feel uncomfortable for a patient with a large craniofacial defect with highly irregular surface contour.

Recently, 3D printers are being utilized to create customized patient‐specific boluses with synthetic materials such as acrylonitrile butadiene styrene (ABS), polylactic acid (PLA), thermoplastic polyurethane (TPU), and polyvinyl acetate.^9^ Compared with milling machines which utilize a drill to carve an object from a solid block of material, the 3D printer is capable of creating a more intricate product with material being added together in a layer‐by‐layer fashion. Several studies have demonstrated the feasibility and accuracy of using 3D printers for fabrication of high‐resolution bolus materials.^10^, ^11^, ^12^, ^13^ The drawback to this new technology is that it is not widely available and the upfront investment in a 3D printer can be fairly expensive. Most printers use rigid synthetic materials; however, Chiu et al described their process of creating customized, soft silicone boluses using a 3D printer. They suggested that for most head and neck cases, an entry level fused deposition modeling (FDM) commercial printer was adequate for use and ranged from $400 to $3000. The time required for production was variable by subsite, but generally could be accomplished in 1 to 2 business days from the time the bolus design was received to delivery.^13^ This system would have the greatest utility in a high‐volume cancer institute, but would be a considerable investment for smaller community practices or institutions that do not capture the populations with the greatest need for customized boluses, such as in head and neck.

Alternatively, we describe an accessible solution for delivering adjuvant radiation to a complex defect of the head and neck using vinylpolysiloxane as bolus material. For years, vinylpolysiloxane has been used as an impression material in the field of prosthodontics, restorative dentistry, and audiology. The material is packaged separately in two components, a base and an accelerator, which can then be mixed together in equal parts. Once mixed together, there are two minutes of working time and 6 minutes of setting time. From a patient perspective, the material is nontoxic, odorless, and tasteless which is advantageous when applied to mucous membranes of the upper aerodigestive tract. Polyvinyl siloxanes are an ideal impression material as they exhibit long‐term dimensional stability and are not susceptible to changes in humidity nor undergo further chemical reactions once set. Most importantly, they are accurate at recording fine surface details and have the best elastic recovery properties compared with other impression materials.^14^, ^15^

It is critical to have a bolus material that conforms accurately to the field of interest as increases in air gaps lead to reduction in the dose delivered to the treatment target.^16^, ^17^ Not only did this impression material serve to minimize air gaps, but it was comfortable enough for the patient to remain in place throughout the duration of his therapy. Unfortunately, the bolus did inadvertently fall out on two occasions which required replacement in clinic. Additionally, the patient lost the prosthetic at one point which required us to make a second prosthetic. The process was easily reproducible and did not incur any significant financial burden. In the future, we could have patients wear something equivalent to a soft‐band to ensure that the prosthetic remains in place. During the day, there were no issues with the prosthetic coming loose or falling out. We would still opt to have the patient keep the device in place at all times during treatment in the future. By circumventing replacing and removing the prosthetic for each treatment session, we avoid distortion of the bolus and further avoid introducing inaccuracies to our radiation delivery system.

The homogeneity and moldable features of vinylpolysiloxane make it an ideal bolus material for delivering therapy to superficial areas of irregular contour in the head and neck region. The cost, simplicity, and efficiency of production using this material offer a distinct advantage over alternative substances or boluses requiring 3D printing. To our knowledge, this case report is the first application of vinylpolysiloxane as a bolus material and represents a unique solution to delivering adjuvant radiation to complex head and neck defects.

## CONFLICT OF INTEREST

The authors declare that there is no conflict of interest regarding the publication of this article.

## AUTHOR CONTRIBUTIONS

All authors were in involved in the writing, revisions, and final review of the manuscript.

## References

[1] Perkins GH , McNeese MD , Antolak JA , Buchholz TA , Strom EA , Hogstrom KR . A custom three‐dimensional electron bolus technique for optimization of postmastectomy irradiation. Int J Radiat Oncol Biol Phys. 2001;51(4):1142‐1151. 11704339 10.1016/s0360-3016(01)01744-8

[2] Kudchadker RJ , Antolak JA , Morrison WH , Wong PF , Hogstrom KR . Utilization of custom electron bolus in head and neck radiotherapy. J Appl Clin Med Phys. 2003;4(4):321‐333. 14604422 10.1120/jacmp.v4i4.2503 PMC5724465

[3] Low DA , Starkschall G , Sherman NE , Bujnowski SW , Ewton JR , Hogstrom KR . Computer‐aided design and fabrication of an electron bolus for treatment of the paraspinal muscles. Int J Radiat Oncol Biol Phys. 1995;33(5):1127‐1138. 7493839 10.1016/0360-3016(95)00257-X

[4] Burleson S , Baker J , Hsia AT , Xu Z . Use of 3D printers to create a patient‐specific 3D bolus for external beam therapy. J Appl Clin Med Phys. 2015;16(3):5247. 26103485 10.1120/jacmp.v16i3.5247 PMC5690114

[5] Su S , Moran K , Robar JL . Design and production of 3D printed bolus for electron radiation therapy. J Appl Clin Med Phys. 2014;15(4):4831. 25207410 10.1120/jacmp.v15i4.4831 PMC5875499

[6] Dubois D , Bice W , Bradford B , Schneid T , Engelmeier R . Moldable tissue equivalent bolus for high‐energy photon and electron therapy. Med Phys. 1996;23(9):1547‐1549. 8892252 10.1118/1.597820

[7] Benoit J , Pruitt AF , Thrall DE . Effect of wetness level on the suitability of wet gauze as a substitute for Superflab as a bolus material for use with 6 mv photons. Vet Radiol Ultrasound. 2009;50(5):555‐559. 19788044 10.1111/j.1740-8261.2009.01573.x

[8] Huang KM , Hsu CH , Jeng SC , Ting LL , Cheng JC , Huang WT . The application of Aquaplast Thermoplastic as a bolus material in the radiotherapy of a patient with classic Kaposi's sarcoma at the lower extremity. Anticancer Res. 2006;26(1B):759‐762. 16739350

[9] Zhao Y , Moran K , Yewondwossen M , et al. Clinical applications of 3‐dimensional printing in radiation therapy. Med Dosim. 2017;42(2):150‐155. 28495033 10.1016/j.meddos.2017.03.001

[10] Zou W , Fisher T , Zhang M , et al. Potential of 3D printing technologies for fabrication of electron bolus and proton compensators. J Appl Clin Med Phys. 2015;16(3):4959. 26103473 10.1120/jacmp.v16i3.4959 PMC5690113

[11] Park JW , Yea JW . Three‐dimensional customized bolus for intensity‐modulated radiotherapy in a patient with Kimura's disease involving the auricle. Cancer Radiother. 2016;20(3):205‐209. 27020714 10.1016/j.canrad.2015.11.003

[12] Łukowiak M , Jezierska K , Boehlke M , et al. Utilization of a 3D printer to fabricate boluses used for electron therapy of skin lesions of the eye canthi. J Appl Clin Med Phys. 2017;18(1):76‐81. 10.1002/acm2.12013 PMC5689892 28291910

[13] Chiu T , Tan J , Brenner M , et al. Three‐dimensional printer‐aided casting of soft, custom silicone boluses (SCSBs) for head and neck radiation therapy. Pract Radiat Oncol. 2018;8(3):e167‐e174. 29452869 10.1016/j.prro.2017.11.001

[14] Chee WW , Donovan TE . Polyvinyl siloxane impression materials: a review of properties and techniques. J Prosthet Dent. 1992;68(5):728‐732. 1432791 10.1016/0022-3913(92)90192-d

[15] Mandikos MN . Polyvinyl siloxane impression materials: an update on clinical use. Aust Dent J. 1998;43(6):428‐434. 9973714 10.1111/j.1834-7819.1998.tb00204.x

[16] Kong M , Holloway L . An investigation of central axis depth dose distribution perturbation due to an air gap between patient and bolus for electron beams. Australas Phys Eng Sci Med. 2007;30(2):111‐119. 17682400 10.1007/BF03178415

[17] Sharma SC , Johnson MW . Surface dose perturbation due to air gap between patient and bolus for electron beams. Med Phys. 1993;20(2 Pt 1):377‐378. 8497226 10.1118/1.597079

